# Evaluation of a chest wall lift technique in feline cadaveric specimens

**DOI:** 10.1111/vsu.14318

**Published:** 2025-08-14

**Authors:** Adrien Aertsens, Emily Humphreys, Tiare Takaesu, Nicholas Rancilio

**Affiliations:** ^1^ College of Veterinary Medicine, Hixson‐Lied Small Animal Hospital Iowa State University Ames Iowa USA; ^2^ Surgery Department Centre Hospitalier Vétérinaire Aquivet Mérignac France

## Abstract

**Objective:**

To evaluate the effect of a simple chest wall‐lifting technique on thoracic volumes in cats.

**Study design:**

Cadaveric study.

**Animals:**

Feline cadavers (*n* = 8).

**Methods:**

Animals underwent thoracic computed tomography (CT) scans after each treatment: in lateral recumbency, (1) no lift and (2) intercostal space lift, and in dorsal recumbency, (1) no lift and (2) sternal lift. Lifting was performed in lateral recumbency at the eighth intercostal space using a bent 2 mm Steinmann pin and in dorsal recumbency at mid‐sternum using a Backhaus towel clamp, placed under thoracoscopic guidance until maximal effect was subjectively noted while recording the tension achieved on the device. Thoracic cavity volume (TCV), working space volume (WSV), and ventilation space volume (VSV) were determined on CT images and compared across treatments.

**Results:**

The TCV increased by a median of 27% (range: 7%–72%), 54% (28%–64%), and 51% (37%–106%) in the dorsal, left lateral, and right lateral recumbencies, respectively. The WSV showed a median increase of 92% (−21%–1026%), 187% (113%–355%), and 271% (119%–422%) for the same positions. In contrast, the VSV decreased, and although statistically significant, the change was relatively minor, with a median decrease of 3% (range: −5%–19%), 7% (−1%–18%), and 7% (3%–11%) in the dorsal, left lateral, and right lateral recumbencies, respectively.

**Conclusion:**

Chest wall lifting increased the WSV in feline cadavers with minimal impact on the VSV.

**Clinical significance:**

This novel and simple technique could help in performing thoracoscopic procedures in cats.

**Abbreviations**
BW: bodyweight
CDI: carbon dioxide insufflation
CT: computed tomography
ICS: intercostal spaces
IPP: intrapleural pressure
OLV: one‐lung ventilation
TCV: thoracic cavity volume
VSV: ventilation space volume
WSV: working space volume

## 1 INTRODUCTION

Video‐assisted thoracoscopic surgery has been increasingly reported in veterinary medicine as a diagnostic or therapeutic procedure.^1^ Sufficient working space is needed to allow proper visualization and instrument manipulation, which is challenging in smaller thoraces.^2^, ^3^ Entering the thoracic cavity induces a pneumothorax which volume can be adequate for some simple thoracoscopic procedures but may be insufficient for more advanced ones.^1^ Two main techniques have been reported to increase the working space volume (WSV) during thoracoscopy in veterinary medicine.^3^ One‐lung ventilation (OLV) is the most popular method but uses mainly human devices whose sizes are inadequate for cats' smaller airways. Double‐lumen endotracheal tubes are too large for small patients, and some endobronchial blockers could fit but guidance with a bronchoscope is challenging.^3^ In one study investigating the use of OLV in cats, the authors concluded that further research is needed before recommending its widespread use in cats.^4^ Intrathoracic carbon dioxide insufflation (CDI) is the other reported technique to increase the WSV. The technique is easy but requires airtight conditions with subsequent limits on the suction use.^5^ To increase the ribcage diameter as seen during inspiration, significant forces are needed and as a result, CDI increases the WSV mainly by compression of the intrathoracic organs rather than displacing outward the ribs and sternum.^6^ The increase in intrathoracic pressure can collapse the lungs, leading to hypoxemia and hypercapnia, while simultaneously elevating central venous pressure and reducing cardiac output, which may result in decreased arterial blood pressure as reported in humans,^7^ horses,^8^ pigs,^9^ dogs,^10^, ^11^ and cats.^2^, ^3^

Anterior chest wall lifting methods have been reported in the human literature in the late 1980s and have been occasionally reported for thoracoscopic management of anterior mediastinal lesions since the late 1990s.^7^ Numerous methods and devices have been reported to lift the body of the sternum, the anterior part of the rib, or the xiphoid process with the patient in dorsal recumbency.^7^ Compared with CDI, the space gained under the sternum with chest wall lifting is reported to be equivalent but without hemodynamic effects and allows suctioning in the surgical field.^5^ Chest wall lifting in lateral recumbency has recently been reported for thoracoscopic procedures in two cats, with one cat showing improved oxygenation and ventilation status upon application of the lift.^12^ Increased thoracic cavity volume (TCV) due to the lift was suspected,^12^ as cats have a compliant chest wall that can yield considerably to external pressure.^13^ We hypothesized that in cats, chest wall lifting in lateral recumbency could improve the working space to a notable extent, thanks to an increase in TCV from lifting not only the upper side of the chest, but by elevating its dependent side off the table. Thus, the purpose of the present study was to evaluate the effects of a simple and practical chest wall lifting method on feline thoracic cavity volumes in lateral recumbencies. While the technique is reported in humans in dorsal recumbency, it was also evaluated in this position.

## 2 MATERIALS AND METHODS

### 2.1 Animals

Feline cadavers euthanized for reason unrelated to the study were used. At the time of euthanasia, these cats were donated by their owners to the Veterinary Teaching Hospital for research and teaching purposes. Per university policy, no institutional animal care and use committee (IACUC) approval was needed for a cadaveric study. Cats with suspected or confirmed thoracic disease at the time of death were excluded. Cadavers were kept frozen at −20°C until completion of the study and thawed at room temperature for 72 h prior to use.

Before the procedure, the thorax was clipped widely. Cuffed endotracheal intubation was performed, and the cat was connected to a semi‐closed breathing system. The cat was mechanically ventilated using a pressure‐controlled ventilator (Mark 7 Respirator, Bird Corp., Palm Springs, California), and continuous positive airway pressure of 5 cm H_2_O was delivered throughout the whole study at a peak inspiratory pressure of 15 cm H_2_O.

### 2.2 Thoracoscopic inspection

Cats were evaluated in dorsal, right lateral and left lateral recumbencies. The order of these positions was randomized using an online randomization program (randomizer.org). All the procedures were performed by one board‐certified surgeon (AA).

A 5.5 mm Thoracoport (Medtronic, Covidien products, Minneapolis, Minnesota) was introduced using standard techniques. A 5.5 mm 30 cm 30° 4 K laparoscope (Arthrex, Inc., Naples, Florida) was inserted through the subxiphoid port when the cat was in dorsal recumbency. In lateral recumbency, the port was placed at the proximal third of the 12th intercostal space. For each recumbency and prior to starting each computed tomography (CT) study, positive airflow >5 cm H_2_O was held through the breathing circuit until proper expansion of the entire lung field was confirmed by thoracoscopic inspection. The endoscope was removed, and the inflation adjusted back to 5 cm H_2_O.

### 2.3 Chest wall lifting device

**FIGURE 1:**
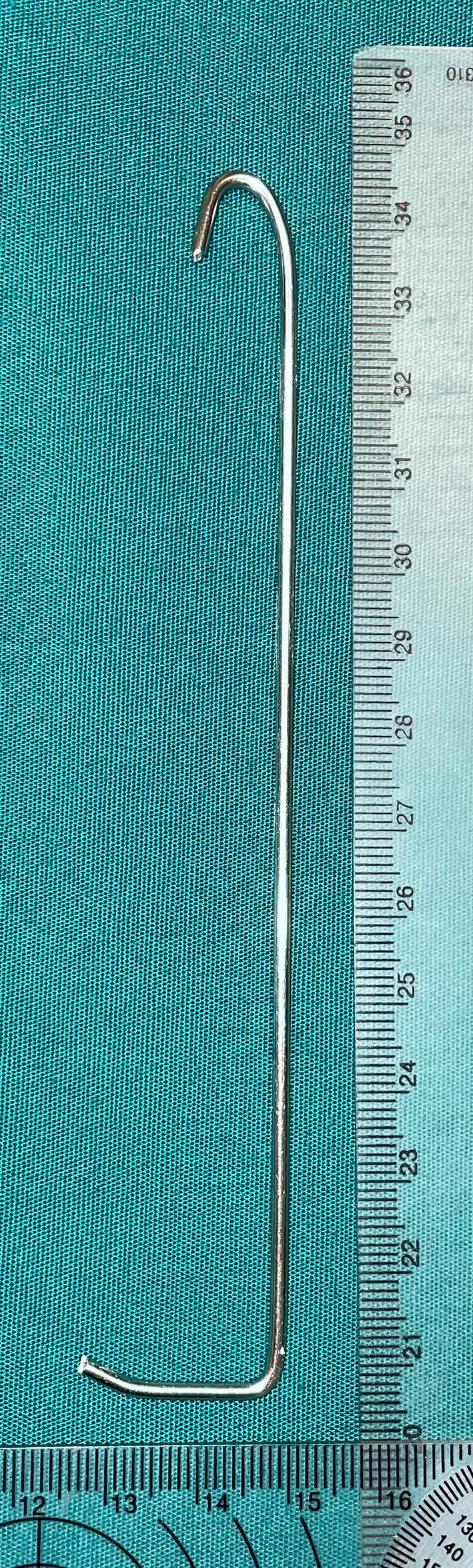
Lift device made of a 2 mm Steinmann pin. The distal portion has a slight dorsal bent to help anchor the pin into the intercostal space and avoid easy pull‐out when lifted. Note the cut tips to decrease the risks of glove perforation and iatrogenic lesions to the intrathoracic structures.

**FIGURE 2:**
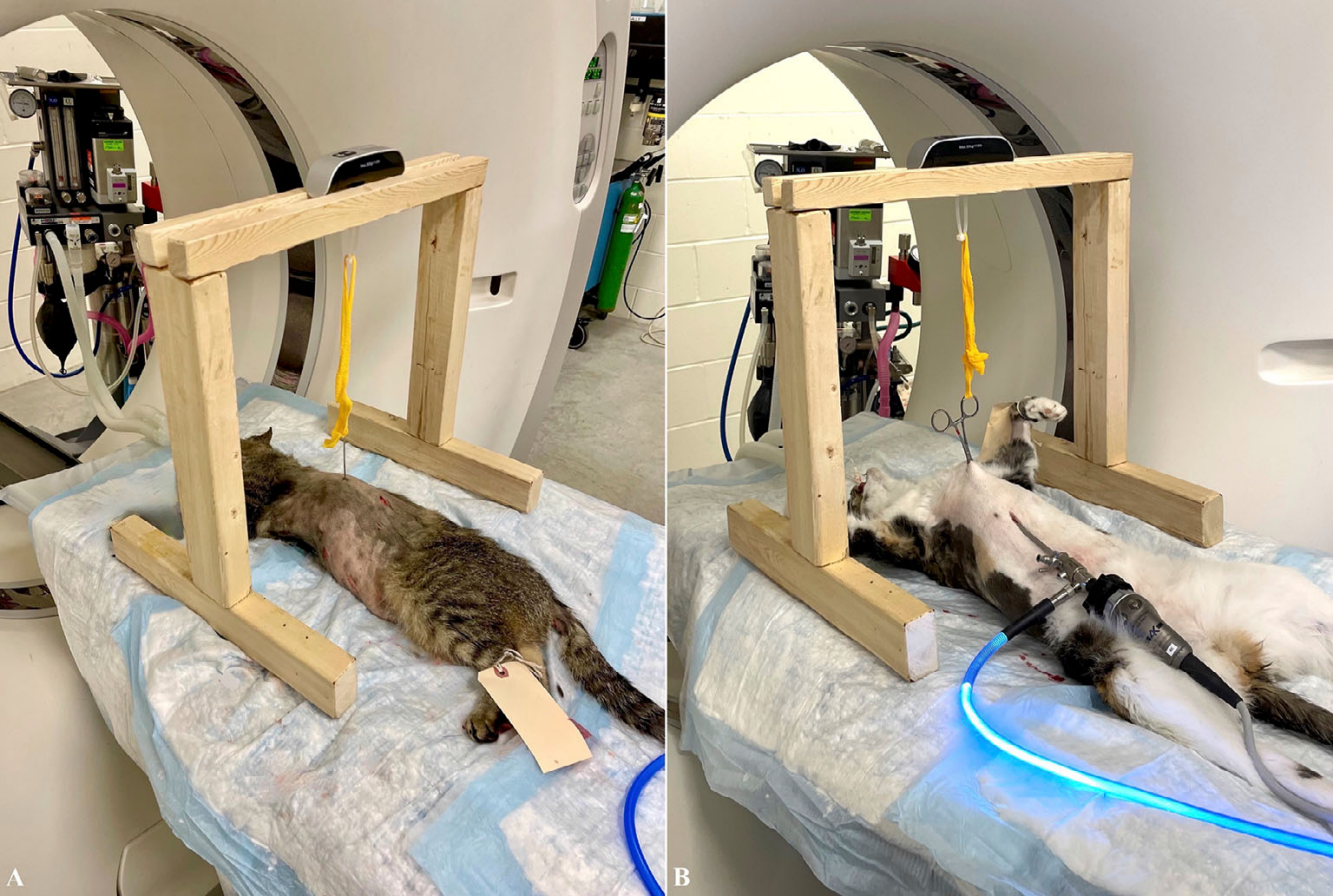
Lift mount with tension measuring device during (A) lateral and (B) dorsal recumbencies. Note how the chest is lifted from the table.

A chest wall lifting device was built using a 2 mm Steinmann pin, bent with conventional Lineman pliers (Lowe's Inc., Mooresville, North Carolina). The size and design of the Steinmann pin were chosen subjectively with the aim of minimizing trauma to the chest wall and lungs while ensuring easy and secure suspension (Figure 1). One side was bent tight at 180° to create a hook for suspension, and the other side was bent at 90° with enough tip length to span two intercostal spaces. This tip was slightly bent upward over 1 cm as it was noted during preliminary trials that this helped holding the device in place. Both tips were cut to minimize risks of iatrogenic trauma. The device was hooked to a strip of 2‐inch self‐adhering cohesive bandage (Vetrap Bandaging Tape, 3M, St Paul, Minnesota), which was anchored to an in‐series digital scale (Portable Electronic Hanging Scale 50 kg/10 g, Electronic Luggage Scale Model EL. 11 by Changzhou Newton Force Weighing Co. Ltd., Jinangsu, China). The maximal tension applied was determined subjectively by monitoring the WSV under endoscopic visualization, and the self‐ adhering cohesive bandage was secured once no further increase in WSV was observed. The lifting tension achieved was recorded for each cat. A rigid custom‐made frame was built to allow hanging of the device while performing the CT images (Figure 2).

Calibration of the digital scales according to the manufacturer was 0.1 kg and was validated in‐house using premeasured weights. The tension applied was verified before and after imaging.

### 2.4 Preliminary study

A preliminary study on two cats was performed to evaluate which intercostal space the lift device should be placed in to induce the most significant WSV gain, and to evaluate if any difference could be found between these spaces. Under thoracoscopic guidance, the lift device was placed at the middle of the fourth, sixth, eighth, and 10th intercostal spaces (ICS) in both lateral recumbencies. During placement, care was taken to keep the tip of the device along the thoracic pleura to avoid iatrogenic lesion to the lungs. A noticeable difference was found between the different locations, with lift at the eighth and 10th ICS leading to the most WSV increase.

In dorsal recumbency, the lift device was placed at the level of the second, fourth and sixth sternebrae, but was challenging to place between the costal cartilages. It was found that by placing a Backhaus towel clamp at the middle of the chest, grasping only the skin and subcutaneous widely, it led to similar gain in WSV. The latter technique was consequently selected as simpler, faster, less traumatic, and less prone to iatrogenic injury.

### 2.5 Procedures

When cats were in lateral recumbencies, a thoracic CT scan (Aquillion 32 Slice; Canon) was performed without any lift. The lift device was then placed under thoracoscopic guidance at the eighth ICS. A second CT was done (Figure 2).

When cats were in dorsal recumbency, the CT scan was performed prior to lifting the skin with a Backhaus towel clamp at the middle of the sternum, and once sternal lift was performed. The lift was monitored with subxiphoid thoracoscopic guidance. (Figure 2).

### 2.6 Volume measurements

**FIGURE 3:**
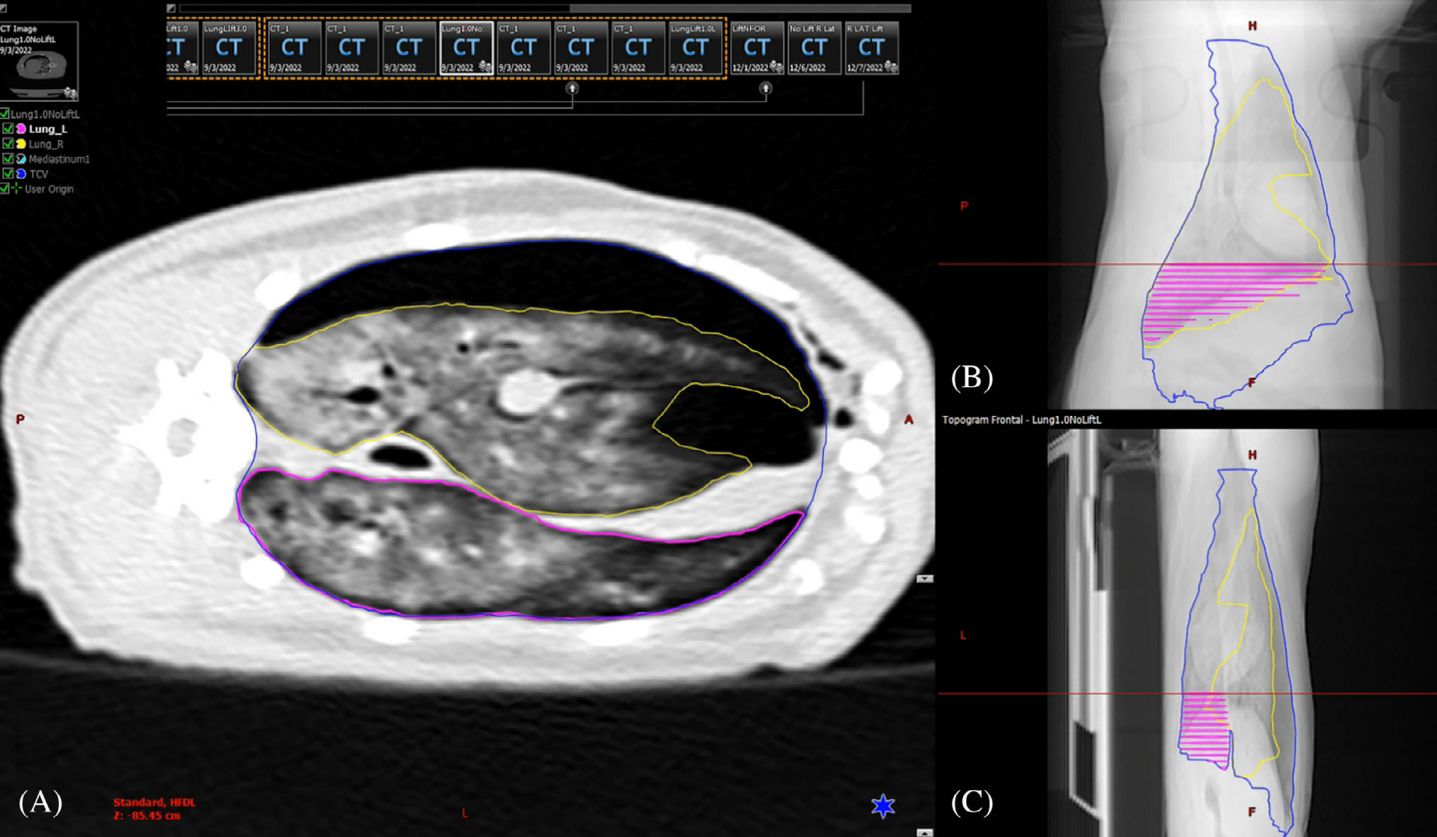
Transverse (A), sagittal (B) and dorsal (C) views of the computed tomography (CT) images of a cat in left lateral recumbency during the volume measurements. In blue, the total cavity volume (TCV) has been contoured. In yellow, the right lung volume has been contoured, and the left lung is contoured in pink. The sum of both lungs' volume is the ventilation space volume (VSV). The working space volume (WSV) is calculated by subtracting the VSV and mediastinal volume (contoured in green) from the TCV.

The TCV was defined as the volume within the parietal pleura. The WSV was defined as the air‐filled region within the thoracic cavity. The ventilated space volume (VSV) was defined as the total lung field, calculated by summing the volume of the right and left lung fields (Figure 3). The volumes were measured by two participants between the thoracic inlet and the diaphragm using Varian Eclipse Radiotherapy Treatment Planning Software (Varian, Palo Alto, California). CT studies were imported to the planning software and the WSV, VSV, and TCV were contoured using automatic and manual tools. All images were reviewed by a board‐certified surgeon and radiation oncologist for anatomic validity prior to calculation. Volumes were compared amongst sequences for each cat and between cats.

### 2.7 Statistical analysis

Descriptive statistics were used to summarize the TCV, WSV, and VSV for the dorsal, left, and right recumbencies before and after lifting. The Wilcoxon signed‐rank test was employed to evaluate the differences in TCV, WSV, and VSV before and after lifting, using a significance level of 0.05. All analyses were conducted using SAS 9.4 (Cary, North Carolina).

## 3 RESULTS

Nine cats were enrolled in the study, but one was excluded because of suspected thoracic disease identified during the thoracoscopy. Of the remaining eight cats, five were females spayed, two were castrated males, and one was an intact male. Median bodyweight (BW) was 4.0 kg (range: 2.6–6.9 kg) and the median body condition score (BCS) was 5/9 (range: 3/9–8/9).

**FIGURE 4:**
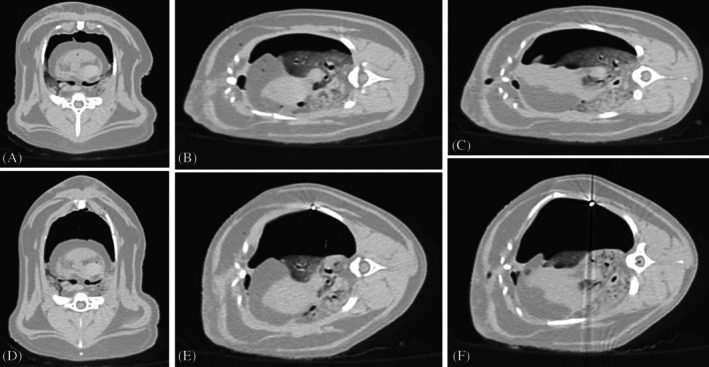
Transverse views of the computed tomography (CT) images of a cat prior (top row) and after (bottom row) chest wall lift, in dorsal (A, D), left lateral (B, E) and right lateral (C, F) recumbencies.

The lift was performed in all cats and in every recumbency (Figure 4). The median tension applied was 0.94 kg (0.27–1.42), 0.88 kg (0.62–1.63) and 0.87 kg (0.57–1.37) in dorsal, left and right lateral recumbencies, respectively. Prior to lifting, the median TCV for the dorsal, left, and right lateral recumbencies were 180.55 mL (137.1–262.2), 188.85 mL (149.6–263.0), and 177.45 mL (144.1–261.0), respectively. Following lifting, these volumes increased significantly to 239.40 mL (200.80–310.30) (*p* = .024), 297.35 mL (245.80–337.70) (*p* < .01), and 279.85 mL (247.60–370.80) (*p* < .01), respectively. (Table 1). The TCV increased by a median of 27% (7%–72%), 54% (28%–64%), and 51% (37%–106%) for the dorsal, left, and right lateral recumbencies, respectively.

**TABLE 1:** Volumes measured prior and after lift.

	Recumbency	Volume before lift (mL) median (min, max)	Volume after lift (mL) median (min, max)	Difference (mL) median (min, max)	*p‐*value
TCV	Dorsal	180.55 (137.10, 262.20)	239.40 (200.80, 310.30)	56.50 (−17.90, 98.50)	.024
	Left lateral	188.85 (149.60, 263.00)	297.35 (245.80, 337.70)	101.65 (72.70, 116.30)	.0078
	Right lateral	177.45 (144.10, 261.00)	279.85 (247.60, 370.80)	103.15 (71.90, 152.70)	.0078
VSV	Dorsal	76.10 (46.60, 116.70)	70.85 (48.80, 116.10)	−2.65 (−16.30, 2.20)	.0391
	Left lateral	69.95 (51.70, 116.90)	61.40 (46.60, 104.00)	−5.20 (−12.90, 0.50)	.0156
	Right lateral	70.25 (52.20, 123.30)	63.65 (50.80, 113.70)	−5.65 (−9.60, −1.40)	.0078
WSV	Dorsal	52.40 (10.20, 128.70)	116.70 (56.80, 180.20)	70.50 (−26.60, 104.70)	.024
	Left lateral	57.25 (26.10, 95.50)	159.65 (118.80, 207.90)	101.75 (86.50, 127.80)	.0078
	Right lateral	51.35 (24.20, 69.90)	155.35 (106.80, 204.30)	112.10 (82.60, 158.80)	.0078

*Note*: *p*‐values were calculated using the Wilcoxon signed‐rank test.

Abbreviations: TCV, total cavity volume; VSV, ventilation space volume; WSV, working space volume.

For the WSV before lifting, median volumes for the dorsal, left and right lateral recumbencies were 52.40 mL (10.20–28.70), 57.25 mL (26.10–95.50) and 51.35 mL (24.20–69.90), respectively. After lifting, these volumes increased significantly to 116.70 mL (56.80–180.20) (*p* = .024), 159.65 mL (118.80–207.90) (*p* < .01) and 155.35 mL (106.80–204.30) (*p* < .01), respectively. (Table 1). The WSV increased by a median of 92% (−21%–1026%), 187% (113%–355%), and 271% (119%–422%) for the dorsal, left, and right lateral recumbencies, respectively.

Before lifting, the median VSV were 76.10 mL (46.60–116.70), 69.95 mL (51.70–116.90) and 70.25 mL (52.20–123.30) for the dorsal, left and right lateral recumbencies respectively. After lifting, the VSV decreased to 70.85 mL (48.80–116.10) (*p* < .05), 61.40 mL (46.60–104.00) (*p* < .05), and 63.65 mL (50.80–113.70) (*p* < .01). (Table 1). The VSV decreased by a median of 3% (−5%–19%), 7% (−1%–18%), and 7% (3%–11%) for the dorsal, left, and right lateral recumbencies, respectively.

## 4 DISCUSSION

Chest wall lifting was easily performed in all cats, causing a significant increase in the TCV and WSV. The gain in working space was more significant in lateral recumbency than in dorsal recumbency.

OLV and CDI have been reported to increase WSV in cats^3^, ^4^ but to the best of our knowledge, there is no study quantifying objectively the gain in working space with these techniques. In dogs in lateral recumbencies, OLV combined with CDI at an intrapleural pressure (IPP) of 3 and 5 mmHg was reported to increase the WSV by 130% and 193%, respectively.^6^ The gain in WSV at 5 mmHg is close to the one achieved using thoracic lift in the present study, but the authors concluded that IPP above 3 mmHg were not recommended considering it decreased the oxygen saturation of the patients.^6^ This has been reported in cats using CDI only, with greater hemodynamic disturbances observed at 5 mmHg compared to 3 mmHg, despite a subjectively assessed similar WSV.^3^ Further studies are needed to objectively compare thoracic volumes prior to and during lift thoracoscopy, OLV, and CDI on cats.

In the present study, the WSV increased more in lateral recumbency than in dorsal recumbency. This finding is not surprising. During inspiration in cats and dogs, the ribs rotate laterally and cranially at the costovertebral joints, which straighten the costal cartilages and cause a caudal displacement of the sternum.^14^, ^15^ As a result, the transverse dimensions of the thorax increase significantly more than the antero‐posterior.^14^, ^15^ Moreover, in lateral recumbency gravity restricts rib motion and consequently expansion of the thoracic wall on the lower side.^16^ When a lifting tension is applied on one side, the ribs are moved laterally at their maximal physiologic motion while the contralateral side is freed from the table and can expand during inspiration, contributing to the increase of TCV and WSV. The opposite was shown in dorsal recumbency; respiratory motion was less affected than in sternal recumbency, with the stiffer spine and proximal part of the ribs restricting the effects of gravity.^16^ When a lifting tension is applied to the sternum, only the sternum is moved ventrally while minimal to no effect is expected on the opposite side lying on the table, leading to less increase in the TCV.

Interestingly, VSV decreased with chest wall lift, and although the change was relatively minor, it was statistically significant. Similarly, this effect was more pronounced in lateral recumbency, which was associated with a larger increase in TCV compared to dorsal recumbency. The stretching of the thoracic wall and changes in the thoracic index induced by the lift may have caused the small thoracoscopic portal to become partially obstructed more rapidly as the lungs expanded, limiting air evacuation from the pneumothorax and potentially leading to partial lung compression. Further research is essential to confirm and clarify the underlying mechanisms, as no anesthetic‐related complications have been reported during sternal lift procedures in humans.^7^ In one of the two feline cases where the technique involving multiple portals has been described, chest wall lift even improved oxygenation and ventilation status.^12^

In a study on lift laparoscopy in dogs, different tensions at different locations were applied successively up to a maximum of 25% of the dog's BW, while simultaneously recording the pneumoperitoneum volumes.^17^ We elected to measure the volumes using a single lifting tension, selected subjectively based on thoracoscopic inspection to achieve the maximal effect, in order to replicate the conditions encountered by surgeons in clinical practice. A wide variation of tension was achieved for every recumbency. This could be secondary to the thawing process, which may have affected chest wall compliance differently in cats with varying BW and BCS, or to the use of the self‐adhering cohesive bandage, which, due to its elastic properties, may have introduced variability in how it was anchored to the scale, despite a standardized technique. Regardless, the median force applied remained below 25% of the BW but it was higher than the 3 kg force required to lift a human chest wall by 3 cm.^7^ Moreover, although lift laparoscopy and lift thoracoscopy are based on the same fundamental principle, their physical and anatomical effects differ significantly. In the peritoneal cavity, port placement alone does not establish working space and the lift technique is used to create one. In contrast, lift thoracoscopy increases the volume of the already existing pneumothorax created by the access port.

In the abdomen, increasing the lifting force induces a tenting effect that is prevented in the chest by the structural support of the thoracic wall.^17^ The local flexibility of the chest wall varies between sites.^18^ During a pilot study, it was found that the maximal effect was achieved when the lifting device was placed at the eighth and 10th intercostal spaces rather than at the fourth and sixth. This finding is not surprising for several reasons. The ribs and costal cartilages length increases proportionally from ribs one to become maximal at ribs nine,^19^, ^20^ contributing to increasing elasticity. The ribs have their greatest transverse movements amplitude from T4 to T11 during spontaneous inspiratory movements in cat.^14^ Lastly, the distal ends of the eighth and ninth ribs are closely associated, articulating with the xiphoid process of the sternum.^19^ This unique sternocostal junction is primarily composed of thick dorsal and ventral sternocostal ligaments, without a synovial joint, unlike the sternocostal joints of the first seven ribs.^19^ At the 10th ribs pairs, the costal cartilage unite to the cartilage of the ninth pairs by connective tissue to form the costal arch.^19^ When distracting significantly the chest wall at the eighth or 10th ICS, elevating the seventh and eighth, and ninth and 10th ribs respectively, these specific anatomic features allow more chest distraction than in the cranial ICS, which move more as a single unit with the sternum.^15^ Based on the findings from the preliminary part of the study, we recommend performing the lift at the eighth, ninth or 10th ICS to optimize the chest wall lift effect, but further studies are needed to confirm this.

Several methods using different devices have been reported to perform sternal lift in the human literature,^7^ but the objective of the authors was to develop a simple and affordable technique, applicable in most veterinary places. A simple option that was initially contemplated was to pass circumcostally a large monofilament suture. This was quickly discarded, because passing completely an appropriately sized needle around the ribs without traumatizing the pulmonary parenchyma is very challenging. Moreover, once the tension is applied, the suture would cut through the intercostal muscles and compress along the ribs the intercostal neurovascular bundle. Kirshner wires used as a lifting device have been reported successfully for sternal lift in human patients with no pain or bruising reported.^21^ Orthopedic pins are cheap and available in most practices offering laparoscopy. We found that the 2 mm Steinman pin offered a good compromise of stiffness and elasticity while remaining small enough for easy insertion between the ribs. Different sizes are available and can be selected according to each patient's BW and can be custom made quickly during the procedure, aiming at spanning two ribs to share the traction forces along the medial side of the thoracic wall.^22^ The sharpness of the tips of orthopedic pins can be improved by cutting these out with a pin cutter, but some sharpness remains and could induce iatrogenic trauma to the patient's lung or the surgeons' gloves during the procedure. Preparing a few pins prior to surgery by sanding down the tips to obtain smooth ends is therefore highly recommended. Bending slightly the intrapleural tip, like the shape of the reported rib or sternum hooks,^23^, ^24^ allows the pin to anchor into the intercostal muscles cranially and avoids slippage during lifting. It eases the insertion of the pin along the pleura but induces some mild trauma into the intercostal muscles. Further studies are needed to evaluate whether the pin design can be improved or if a dedicated device could be developed to minimize iatrogenic trauma while facilitating easier insertion and removal.

In dorsal recumbency, various methods have been reported in the human literature for chest wall lifting at the sternum body, xiphoid process or anterior part of the ribs.^7^ Clamping the skin with a towel clamp was found to be as effective as placing a pin dorsal to the sternum, while reducing trauma and the risk of severe iatrogenic trauma. Sternal lift using a pin passed through the subcutaneous tissue only has been reported as being efficient and safe in the human literature,^21^ but a study on live cats is needed to confirm this as the skin and subcutaneous tissue elasticity may have been affected by the freezing and thawing process, affecting our findings.

The technique has several disadvantages. The primary concern is iatrogenic trauma to the intrathoracic structures during pin manipulation especially when compared to the safer techniques of OLV or CDI. No obvious iatrogenic lesions were identified during the study; however, the tissue in cadavers may not behave similarly to that in a living cat. A hanging tool, either from the roof or anchored reliably to the operating table, is needed for the lifting procedure and can obscure the view of thoracoscope monitor. Lastly, the technique may not be feasible on every patient. Cats have higher respiratory system compliance than dogs,^25^ but this decreases with growth and development.^26^, ^27^ Additionally, the chest wall compliance in older or diseased cats may limit the potential gain in working space while increasing pain.^26^, ^27^

The study had several very important limitations. A major limitation was the cadaveric nature of the project, which cannot emulate the challenges on live animals with intrathoracic disease. Effects on ventilation and pain could not be evaluated. A small cohort of only eight cadavers was used and different complications may have occurred had a larger cohort of cats been used. No comparison to CDI was made, precluding interpretation of the WSV gain. Other limitations of the technique include unknown repeatability of the device creation and repeated use of the same device throughout the study.

Chest wall lifting is feasible in cats and significantly improves the working space for thoracoscopic procedures, especially in lateral recumbency. Lift could be performed at the eighth, ninth or 10th ICS to optimize the effect while minimizing the number of ports if the lifting device is placed next to a port through the same incision. This minimally invasive technique might be considered as an auxiliary means to increase the feasibility and utility of thoracoscopy in cats.

## AUTHOR CONTRIBUTIONS

Aertsens A, DVM, MRCVS, DECVS: Designed the study, performed the procedures, drafted and revised the manuscript. Humphreys E: Contributed to the procedures, performed the measurements and revised the manuscript. Takaesu T: Contributed to the procedures, performed the measurements and revised the manuscript. Rancilio N, DVM, MS, DACVR (Radiation Oncology): Contributed to the design of the study, oversaw data collection and revised the manuscript. All authors provided a critical review of the manuscript and endorse the final version. All authors are aware of their respective contributions and have confidence in the integrity of all contributions.

## CONFLICT OF INTEREST STATEMENT

The authors have no conflict of interest to declare.
